# Study of Rolling Motion of Ships in Random Beam Seas with Nonlinear Restoring Moment and Damping Effects Using Neuroevolutionary Technique

**DOI:** 10.3390/ma15020674

**Published:** 2022-01-17

**Authors:** Naveed Ahmad Khan, Muhammad Sulaiman, Carlos Andrés Tavera Romero, Ghaylen Laouini, Fahad Sameer Alshammari

**Affiliations:** 1 Department of Mathematics, Abdul Wali Khan University, Mardan 23200, Pakistan; ahmednaveed854477@gmail.com; 2 COMBA R&D Laboratory, Faculty of Engineering, Universidad Santiago de Cali, Cali 76001, Colombia; carlos.tavera00@usc.edu.co; 3 College of Engineering and Technology, American University of the Middle East, Egaila 54200, Kuwait; ghaylen.laouini@aum.edu.kw; 4 Department of Mathematics, College of Science and Humanities in Alkharj, Prince Sattam bin Abdulaziz University, Al-Kharj 11942, Saudi Arabia

**Keywords:** steady-state roll motion, nonlinear damping, random beam seas, artificial neural networks, Levenberg-Marquardt algorithm, soft computing

## Abstract

In this paper, a mathematical model for the rolling motion of ships in random beam seas has been investigated. The ships’ steady-state rolling motion with a nonlinear restoring moment and damping effect is modeled by the nonlinear second-order differential equation. Furthermore, an artificial neural network (NN)-based, backpropagated Levenberg-Marquardt (LM) algorithm is utilized to interpret a numerical solution for the roll angle (x(t)), velocity $\left(x^{\prime }\left(t\right)\right)$, and acceleration $\left(x^{\prime \prime }\left(t\right)\right)$ of the ship in random beam seas. A reference data set based on numerical examples of the mathematical model for a rolling ship for the LM-NN algorithm is generated by the numerical solver Runge–Kutta method of order 4 (RK-4). The LM-NN algorithm further uses the created data set for the validation, testing, and training of approximate solutions. The outcomes of the design paradigm are compared with those of the homotopy perturbation method (HPM), optimal homotopy analysis method (OHAM), and RK-4. Statistical analyses of the mean square error (MSE), regression, error histograms, proportional performance, and computational complexity further validate the worth of the LM-NN algorithm.

## 1. Introduction

**Figure 1:**
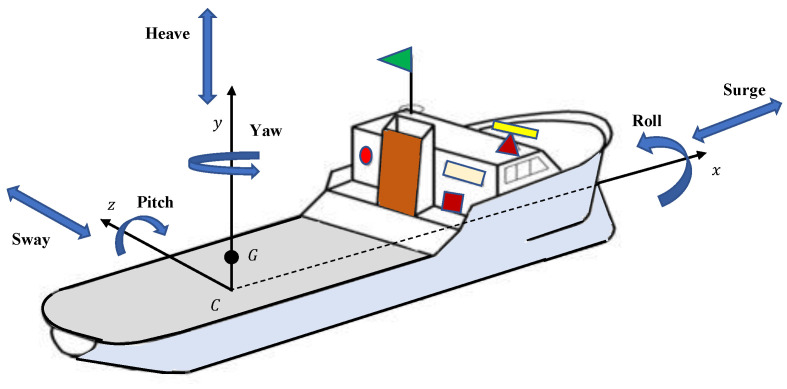
Schematic diagram of ship showing six directions of motion.

In general, ships experience different motions, including angular and displacement motions, categorized as yaw, pitch, roll, heave, drift, and surge. Figure 1 represents the schematic directions of all six motions. The stabilization of a ship depends on two methods, namely roll reduction and the modeling or evaluation method of roll performance. In the later part of the mid-18th century, Froude studied a ship’s rolling motion for the first time. Later on, Norio Tanaka [1] introduced the empirical and semi-empirical roll damping coefficient for dynamic equations. A simple method was proposed by Himeno [2] for the prediction of the roll damping of ships at forwarding speed. In 2004, Ikeda [3] presented the modified model of roll damping with a steady drift motion.

In sea studies, roll damping is one of the important topics of discussion for most researchers. However, the complexity of problems and the potential flaws in traditional techniques make the solving of roll motion problems difficult. Scientists have adopted various strategies, including empirical formulas, advanced experimental processes, computational fluid dynamics (CDF), an analysis method for roll dumping, a finite differential model, and a higher polynomial roll dumping model. Yeung [4] implemented a method based on CDF that had been used for local phenomena of vortex shedding around bilge keels. An experimental analysis on the local flow visualization of roll damping are given in [5,6,7].

Researchers have recently focused on studying the mathematical modeling of the roll damping of ships. It has been noticed that external forces significantly affect ship stability, which causes complexity in mathematical models. The strong nonlinear terms arising in the mathematical model make it possible to study the behavior of forecasting stability changes in operations [8]. Oliveira and Fernandes [9] used the hyperbola or bilinear fitting method, and Agarwal [10], in 2015, used a fractional differential equation model to investigate the roll damping phenomena of ships. The ship’s rolling motion in terms of a second-order nonlinear differential equation is of great significance to the research community. Finding a closed-form solution (exact solution) to such a model is difficult. Various numerical and perturbation techniques have been implemented to find the approximate solutions. Some well-established techniques for obtaining analytical expressions for roll angle, velocity, and acceleration are the finite element method (FEM) [11], differential transformation method (DTM) [12], homotopy analysis method (HAM) [13,14,15], variational iteration method (VIM) [16,17], homotopy perturbation method (HPM) [18], modified homotopy perturbation method [19], Green function-based method (GFM) [20], series method (SM) [21], and the ultraspherical wavelets-based method (UWM) [19]. Although these methods have advantages, especially over non-typical appendages in size and shape and newer hull designs, most of the methods are gradient-based techniques and depend on traditional deterministic approaches that have not been validated.

In recent times, stochastic optimization techniques based on artificial neural networks have been adopted to find numerical solutions to various complex and stiff problems. Recently, such soft computing techniques have been applied to study the wire coating phenomena [22], diabetic retinopathy classification using fundus images [23], imbibition phenomena [24], heat transfer in porous fins [25,26,27], wire coating dynamics [22], beam-column designs by varying axial load [28], absorption of carbon dioxide $\left(CO_{2}\right)$ into solutions of phenyl glycidyl ether [29], mathematical models of CBSC over wireless channels [30], Michaelis–Menten kinetics in a micro-disk biosensor [31], a mathematical model for eye surgery [32], and electrohydrodynamic flow in a circular cylindrical conduit [33]. The techniques mentioned above have motivated the authors of this study to numerically solve the mathematical model of the ship’s rolling motion by using a stochastic Levenberg-Marquardt (LM) algorithm based on neural networks that have never been applied to such a model. The potential outcomes of the presented work are summarized as follows:

- The formulation of a mathematical model for the rolling motion of ships in random beam seas will have been investigated;
- The novel, integrated design of a computing paradigm based on the two-layer structure of the Levenberg-Marquardt (LM) algorithm and neural networks (LM-NNs) is presented to examine the rolling motions;
- The model is briefly analyzed by considering certain examples depending on variations in angular frequency $\left(\omega_{0}^{2}\right)$, damping coefficient (μ), frequency (ω), amplitude (ϵ), and strength of nonlinearity coefficient (α);
- A merit function based on the mean square error is effectively developed for the computational analysis of LM-NNs by taking reference solutions of different examples generated by the RK-4 method;
- The training, testing, and validation process of LM-NNs are utilized to study the performance of approximate solutions by graphically illustrating regressions, absolute errors, and error histograms. The results of LM-NNs are compared with those of the HPM and RK-4 methods, which shows the dominance of the technique;
- An advantage of the proposed design is that it does not require any initial parameter settings. Its implementation is simple and smooth, with exhaustive applicability and stability.

## 2. Problem Formulation

In 1981, Cardo [34] formulated the mathematical equation for the rolling motion of a ship without any influence from oscillation, which is given as follows:

(1) $$I\ddot{\xi }+M_{r}\left(\Theta ,t\right)+D\left(\Theta ,\dot{\Theta }\right)=E_{W}\cos\Omega t,$$

Equation (1) represents the general equation of the roll motion of ships in the absolute heeling angle, where ξ, $\dot{\xi }$, and $\ddot{\xi }$ denote the roll angle, velocity, and acceleration, respectively. The moment of inertia is denoted by *I*, *D* denotes the moment of forces, $E_{w}$ is amplitude, $M_{r}$ is the righting moment, and ω is the angular frequency. Here, the nonlinear damping term is taken into consideration as the angular dependence of the linear term, which is given by the following equation:

(2) $$D\left(\xi ,\dot{\xi }\right)=\left(D_{01}+D_{21}\xi^{2}\right)\dot{\xi }+D_{03}{\dot{\xi }}^{3},$$

its normalized form of the righting moment can be expressed as:

(3) $$M_{r}^{*}\left(\xi \right)=\omega_{0}^{2}\xi +\alpha_{3}\xi^{3},$$

establishing dimensionless parameters by introducing the time $T_{n}$ and angle $\xi_{n}$ scales as follows:

$$\tau =\frac{t}{T_{n}},\;x=\frac{\xi }{\xi_{n}},\;\omega =\Omega T_{n},\;\omega_{0}^{2}=\frac{\Delta k_{1}T_{n}^{2}}{I},\;\delta_{2}=\frac{D_{03}\phi_{n}^{2}}{IT_{n}},$$

$$\alpha_{i}=\Delta k_{i}T_{n}^{2}\xi_{n}^{i-1},\;\delta_{1}=\frac{D_{21}T_{n}\xi_{n}^{2}}{I},\;\mu =\frac{D_{01}T_{n}}{2I},\;\varepsilon =\frac{E_{w}T_{n}^{2}}{I\phi_{n}},$$

using the above parameters and rearranging the term gives the nonlinear equation of the cubic damping moment [34]:

(4) $$\ddot{x}+\left(2\mu +\delta_{1}x^{2}\right)\dot{x}+\delta_{2}x^{3}+\omega_{0}^{2}x+\alpha_{3}x^{3}=\varepsilon \cos\left(\omega t\right).$$

where α, μ, ε, and ω denote the strength of nonlinearity, damping coefficient, amplitude, and angular frequency, respectively. $\delta_{1}$ and $\delta_{2}$ are viscous damping coefficients.

## 3. Proposed Methodology and Performance Indices

### 3.1. Structure of Artificial Neural Networks

Artificial neural networks (ANNs) are intelligent computational systems that mimic the biological nervous system. ANNs have been successfully applied by a number of researchers to study the complex problems such as pattern identification, recognition, classification [35], electrical energy consumption forecasting [36], and induction motor drive in pumping [37].

**Figure 2:**
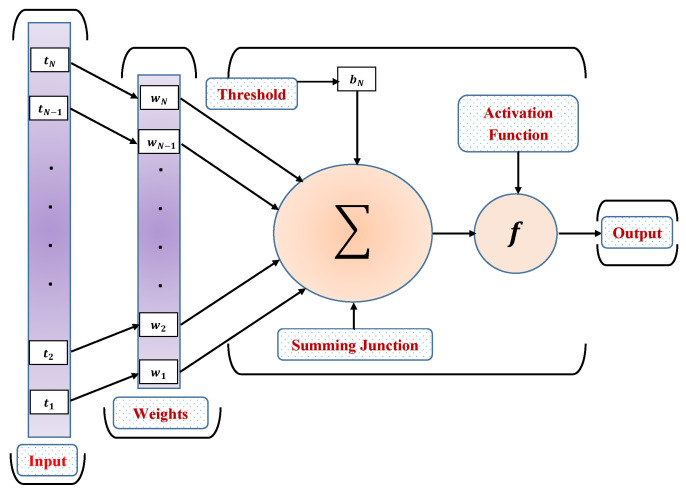
Basic structure of a simple artificial neuron.

The fundamental structure of ANNs comprises of interconnected neurons and nodes that receives the input, combines them in a specific way, and performs some nonlinear operation to generate the output. Figure 2 depicts the architecture of ANNs that consists of input, weight, threshold, summing junction, and output. For the basic model of ANNs, the input $t_{N}$ is multiplied by the connection weights, and the bias or threshold is further applied to convert the inputs into the desired results. The net input is calculated as follows:

(5) $$u_{k}=\sum_{k=1}^{N}w_{k}t_{k}-b_{k}.$$

In order to generate the output x(t), an activation function such as Log-Sigmoid is used, which is given as:

(6) $$x_{k}=f\left(\sum_{k=1}^{N}w_{k}t_{k}-B_{k}\right),$$

where *N* is the number of inputs.

### 3.2. Learning Procedure

**Figure 3:**
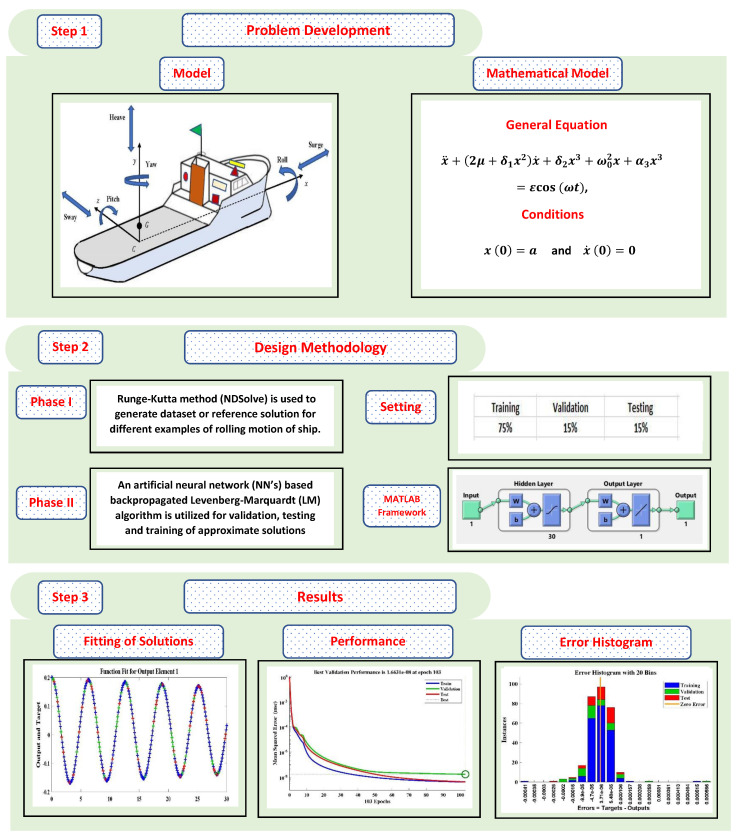
Architecture of the proposed methodology of the mathematical model for the rolling motion of ships in random beam seas.

**Figure 4:**
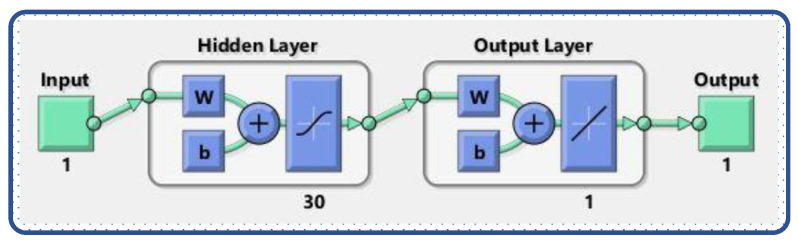
Structure of the supervised neural network [38].

In this section, the learning procedure of the designed weights in the ANN structure is discussed. The implementation of the novel design of the Levenberg-Marquardt neural network approach is based on two steps. In the first step, a mathematical model for the rolling motion of the ship is evaluated by the Runge–Kutta method of order 4 using “NDSolve”, the built in function of Mathematica to generate the reference solution of 301 data points, with a 0.1 step size from 0 to 30. In the second step, the Levenberg-Marquardt technique, which is an efficient technique in the field of soft computing, is implemented using the “nftool” routine on MATLAB for the proper training, validation, and testing of the problem. The work flow and parameter settings in terms of training, validation, and testing for the LM-NN algorithm is shown in Figure 3. The computational model with a double neural network for the design scheme has been plotted through Figure 4.

The performance of the designed scheme is measured through the performance indicators in terms of the mean square error (MSE) of the fitness function of the model, regression $R^{2}$, error histograms, and absolute errors (AE). The mathematical formulation of the MSE, $R^{2}$, and AE are given as follows:

(7) $$\mathrm{MSE}=\frac{1}{k}\sum_{j=1}^{k}{\left(x_{j}\left(t\right)-{\hat{x}}_{j}\left(t\right)\right)}^{2},$$

(8) $$R^{2}=1-\frac{\sum_{j=1}^{k}{\left({\hat{x}}_{j}\left(t\right)-{\bar{x}}_{j}\left(t\right)\right)}^{2}}{\sum_{j=1}^{k}{\left(x_{j}\left(t\right)-{\bar{x}}_{j}\left(t\right)\right)}^{2}},$$

and

(9) $$\mathrm{AE}=\left|x_{j}\left(t\right)-{\hat{x}}_{j}\left(t\right)\right|,\;j=1,2,\ldots ,k,$$

where $x_{j}$, ${\bar{x}}_{j}$, and ${\hat{x}}_{j}$ denote the reference, approximate, and mean of the solution at the *j*th input, and *k* is the number of mesh points. The desired value for the MSE and AE for perfect fitting is equal to zero, while the value of $R^{2}$ is one.

## 4. Numerical Experimentation

**Figure 5:**
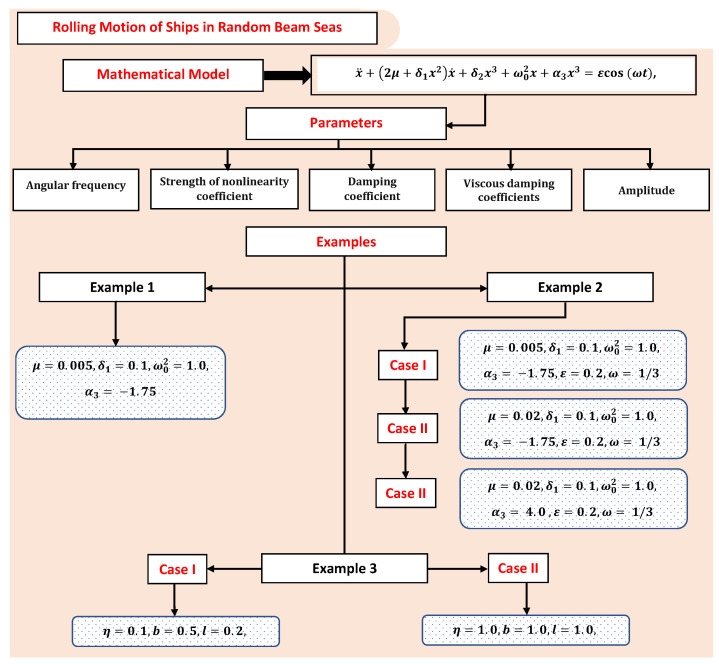
An overview of different examples and cases studied in this paper.

In this section, we considered certain examples of the rolling motion of ships in random beam seas by varying certain parameters. Figure 5 shows the flow chart of the problems discussed in this paper.

**Example** **1.** *In this example, a nonlinear differential equation for the rolling motion of ships is considered [19,34]:*

(10) $$\ddot{x}+\left(2\mu +\delta_{1}x^{2}\right)\dot{x}+\omega_{0}^{2}x+\alpha_{3}x^{3}=0,$$

*subjected to*

(11) $$x\left(0\right)=a\;and\;\dot{x}\left(0\right)=0.$$

Experimental values of the parameters involved in Equation (10) are $\delta_{1}=0.1$, μ=0.005, $\alpha_{3}=-1.75$, a=0.2, and $\omega_{0}^{2}=1$.

**Example** **2.** *In this example, a nonlinear IVP of the cubic damping moment of the rolling motion is considered [34], which is given as follows:*

(12) $$\ddot{x}+\left(2\mu +\delta_{1}x^{2}\right)\dot{x}+\omega_{0}^{2}x+\alpha_{3}x^{3}=\varepsilon \cos\left(\omega t\right),$$

*with the initial conditions*

(13) $$x\left(0\right)=0.2\;and\;\dot{x}\left(0\right)=0.$$

Furthermore, to briefly study the model, the following three cases are considered, depending on variations in the dimensionless damping coefficient and the strength of the nonlinearity coefficient. Case I: $\delta_{1}=0.1$, μ=0.005, $\alpha_{3}=-1.75$, $\omega_{0}^{2}=1$, ε=0.2, and ω=0.333333. Case II: $\delta_{1}=0.1$, μ=0.02, $\alpha_{3}=-1.75$, $\omega_{0}^{2}=1$, ε=0.2, and ω=0.333333. Case III: $\delta_{1}=0.1$, μ=0.02, $\alpha_{3}=4.0$, $\omega_{0}^{2}=1$, ε=0.2, and ω=0.333333.

**Example** **3.** *In this example, we considered the cubic damping moment of the nonlinear roll motion [14,39], which is given by the following equation:*

(14) $$\ddot{x}\left(1+4b^{2}x^{2}\right)+\eta x+4b^{2}x^{2}x=0,\;\eta >0,$$

*with*

(15) $$x\left(0\right)=l\;and\;\dot{x}\left(0\right)=0.$$

Two cases of Equation (14) are considered. Case I: b=0.5, η=0.1, and l=0.2. Case II: b=1.0, η=1.0, and l=1.0.

### Chaos Phenomena of Ship Nonlinear Rolling Motion

When a ship is sailing, the motion of the ship is extremely complex. However, the large restoring and nonlinear torques cannot be ignored. AH Nayfeh [40], in 1990, took nonlinear damping and restoring torques into consideration and investigated the complexity and stability of the dynamics in the rolling motion of ships under the influence of different slopes and wave surfaces. The mathematical model for the nonlinear motion of ships subjected to the regular waves can be written as follows [41,42]:

(16) $$\left(I+I^{*}\right)\ddot{\xi }+D\left(\dot{\xi }\right)+M\left(\xi \right)=B_{1}-M\left(\dot{\alpha },\ddot{\alpha }\right),$$

where $M(\dot{\alpha },\ddot{\alpha })$ represents the wave disturbance torque, and α denotes the slope or gradient of the wave surfaces and is defined as:

(17) $$\alpha =\alpha_{m}\cos\left(\omega t\right),$$

here, $\alpha_{m}$ is the maximum slope. The chaotic phenomena are extremely sensitive, particulary to the external disturbances, therefore the $M(\dot{\alpha },\ddot{\alpha })$ can be given as follows:

(18) $$M\left(\dot{\alpha },\ddot{\alpha }\right)=-f_{1}\alpha_{m}\omega \sin\omega t-f_{2}\alpha_{m}\omega^{2}\cos\omega t,$$

where $f_{1}\alpha_{m}\omega \sin\omega t$ and $f_{2}\alpha_{m}\omega^{2}\cos\omega t$ are the restoring and damping disturbance torques. Equation (17) is reduced to the following equation:

(19) $$\left(I+I^{*}\right)\ddot{\xi }+D\left(\dot{\xi }\right)+M\left(\xi \right)=B_{1}+f_{1}\alpha_{m}\omega \sin\omega t+f_{2}\alpha_{m}\omega^{2}\cos\omega t,$$

$D(\dot{\xi })$ and M(ξ) can be given as:

(20) $$D\left(\dot{\xi }\right)=2\mu_{1}\dot{\xi }+\mu_{3}{\dot{\xi }}^{3},$$

(21) $$M\left(\xi \right)=\omega_{0}^{2}\xi +\alpha_{3}\xi^{3}+\alpha_{5}\xi^{5}+\ldots ,$$

here, $2\mu_{1}$ and $\mu_{3}$ are linear and cubic damping coefficients of torque. Moreover, $\alpha_{3}$, $\alpha_{5}$ are constants and $\omega_{0}^{2}$ is the linear restoring coefficient of torque. $B_{1}$ is constant, and its value can be calculated as follows:

(22) $$B_{1}=\omega_{0}\xi_{s}+\alpha_{3}\xi_{s}+\alpha_{5}\xi_{s},$$

where $\xi_{s}$ represents the heeling angle. Using Equations (20)–(22) in Equation (19) will result in the following:

(23) $$\left(I+I^{*}\right)\ddot{\xi }+2\mu_{1}\dot{\xi }+\mu_{3}{\dot{\xi }}^{3}+\omega_{0}^{2}\xi +\alpha_{3}\xi^{3}+\alpha_{5}\xi^{5}=f_{1}\alpha_{m}\omega \sin\omega t+f_{2}\alpha_{m}\omega^{2}\cos\omega t.$$

The mathematical model is simplified by introducing dimensionless parameters such as:

(24) $$m_{1}=\frac{2\mu_{1}}{I+I^{*}},m_{2}=\frac{\mu_{3}}{I+I^{*}},n_{1}=\frac{\omega_{0}^{2}}{I+I^{*}},n_{2}=\frac{\alpha_{3}}{I+I^{*}},n_{3}=\frac{\alpha_{5}}{I+I^{*}},A_{1}=\frac{f_{1}}{I+I^{*}},\;\mathrm{and}\;A_{2}=\frac{f_{2}}{I+I^{*}}.$$

Equation (23) can be given as follows:

(25) $$\ddot{\xi }+m_{1}\dot{\xi }+m_{2}{\dot{\xi }}^{3}+n_{1}\xi +n_{2}\xi^{3}+n_{3}\xi^{5}=A_{1}\alpha_{m}\omega \sin\omega t+A_{2}\alpha_{m}\omega^{2}\cos\omega t.$$

Values of the parameters involved in Equation (25) are given in Table 1. One of the primary characteristics of a chaotic system is that there is great sensitivity to the initial values. The traces formed by the small difference between the two initial values will disperse in the usual way as time goes on [43]. Equation (25) is similar to duffing equations, which are strongly nonlinear and are commonly used for detecting the weak signals in the chaotic systems. Furthermore, to study the chaos phenomena in ships, Equation (25) is reduced to the system of differential equations by letting $x=\xi ,y=\dot{\xi }$, and z=ωt:

(26) $$\left\{\begin{array}{c} \dot{x}-y=0,\\ \dot{y}=-3.240x+4.5250x^{3}-0.8780x^{5}-0.3500y-0.0222y^{3}+0.5040\alpha_{m}\omega \sin z-4.6656\alpha_{m}\omega^{2}\cos z,\\ \dot{z}-\omega =0, \end{array}\right.$$

with the initial conditions

(27) x(0)=y(0)=0,andz(0)=1.

## 5. Results and Discussion

**Figure 6:**
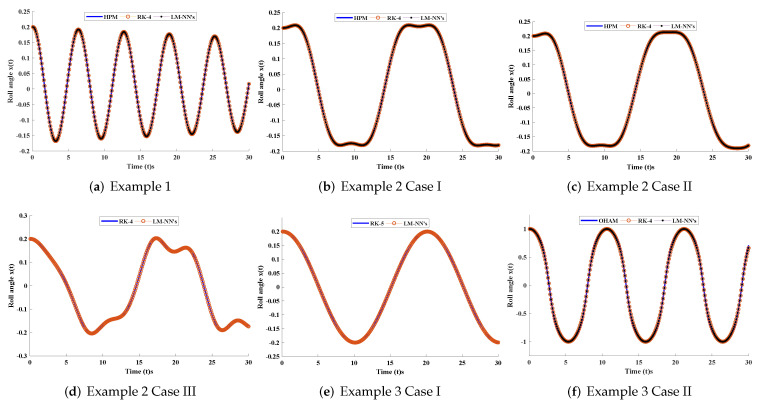
Comparison of approximate solutions with the numerical solver (RK-4), HPM, and OHAM for different examples of the rolling motion of ships.

**Figure 7:**
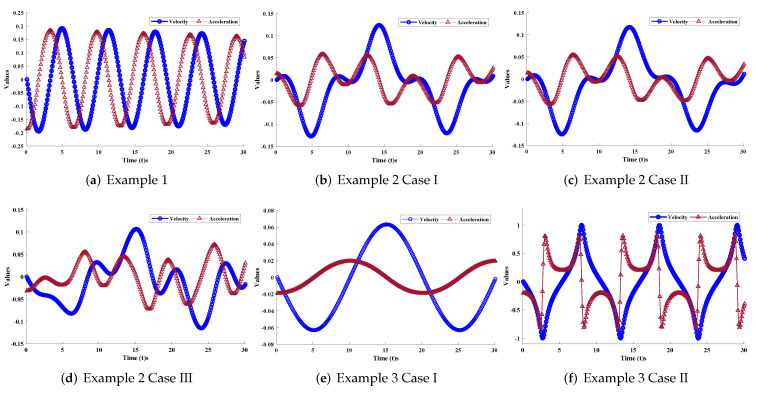
Plots of velocity $\dot{x}\left(t\right)$ and acceleration $\ddot{x}\left(t\right)$ for different cases of the cubic damping motion of ships in random beam seas.

**Figure 8:**
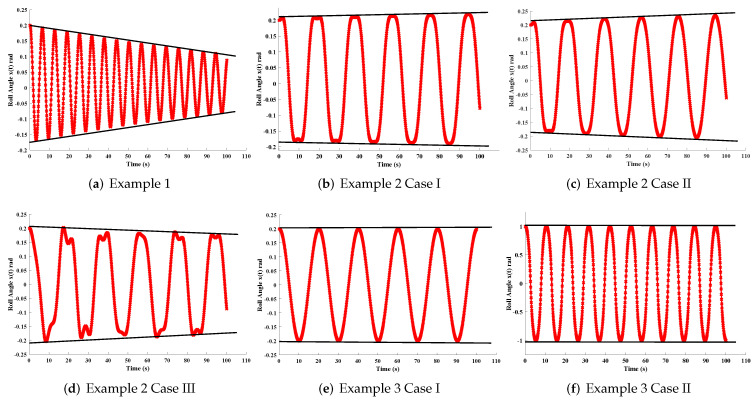
Roll angle decay curves for each example.

**Figure 9:**
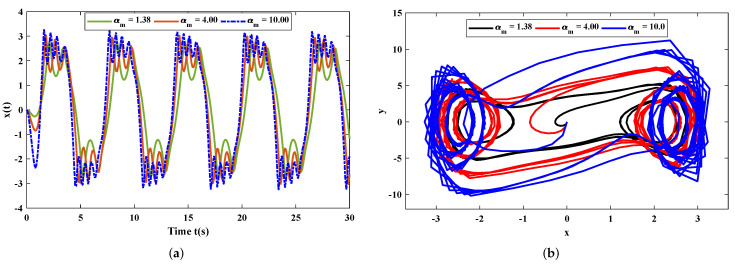
(**a**) Demonstrates the influence of variations in $\alpha_{m}$ on the rolling motion of ships. (**b**) Phase-space diagram of y(t) against x(t) for different values of the maximum slope.

In this section, the implementation of the proposed LM-NN algorithm to solve the mathematical model for the rolling motion of ships in random beam seas is discussed. Numerical and graphical results are illustrated for all three mathematical examples with different cases. An approximate solution for each example obtained by the LM-NNs is compared with those of the homotopy perturbation method (HPM) [19], the Runge–Kutta method (RK-4), and the optimal homotopy analysis method (OHAM) [39], as shown in Figure 6. Table 2 and Table 3 represent the statistics of the obtained values for the rolling angle, velocity, and acceleration. The behaviour of variations in different parameters on $\dot{x}\left(t\right)$ and $\ddot{x}\left(t\right)$ are shown in Figure 7. Figure 8 illustrates rolling decay curves that show the impact of time on the rolling angle. Furthermore, to study the chaotic behaviour of the rolling motion of ships, Equation (26) is solved by the LM-NNs to study the influence of variation on maximum slope $\alpha_{m}$. Figure 9a shows the frequency spectrum diagram of the motion. It can be observed that increasing the value of $\alpha_{m}$ causes an increase in the rolling angle of the ship. The system’s dispersion phenomena become obvious, and the rolling angle of the ship’s motion increases correspondingly. Figure 9a analyzes the chaotic behaviour of the rolling motion of ships.

**Figure 10:**
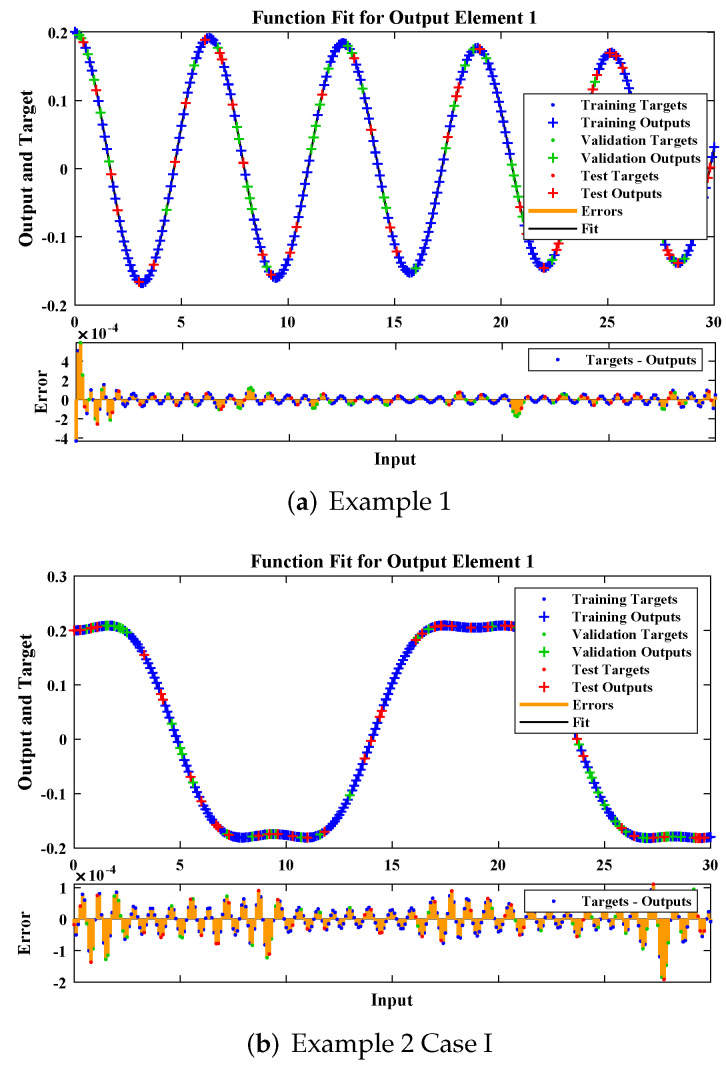

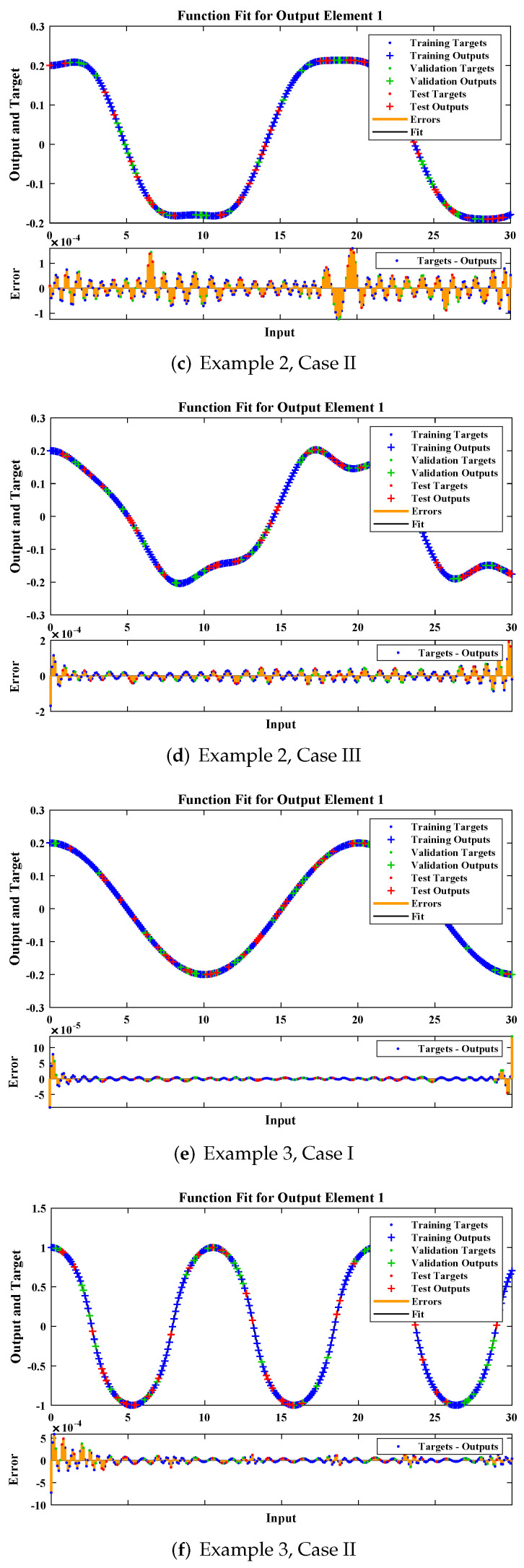
Analysis of the fitness plot by the LM-NN algorithm for different examples.

**Figure 11:**
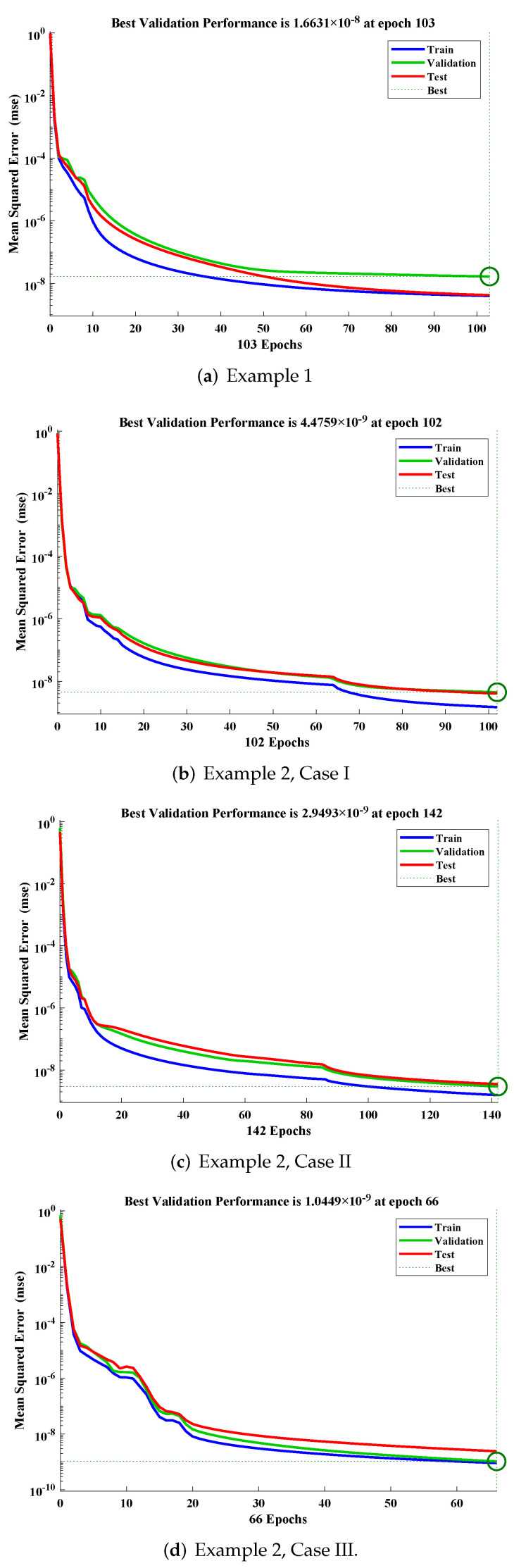

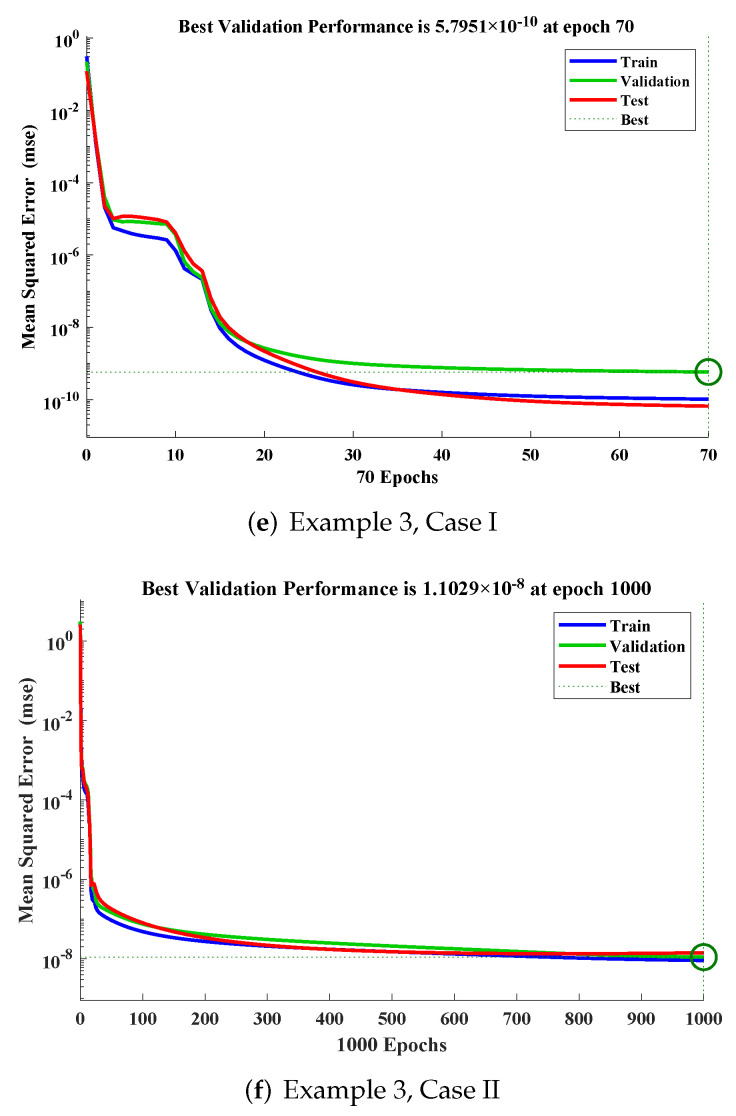
Studies based on the performance of MSE by the design scheme, for multiple examples.

**Figure 12:**
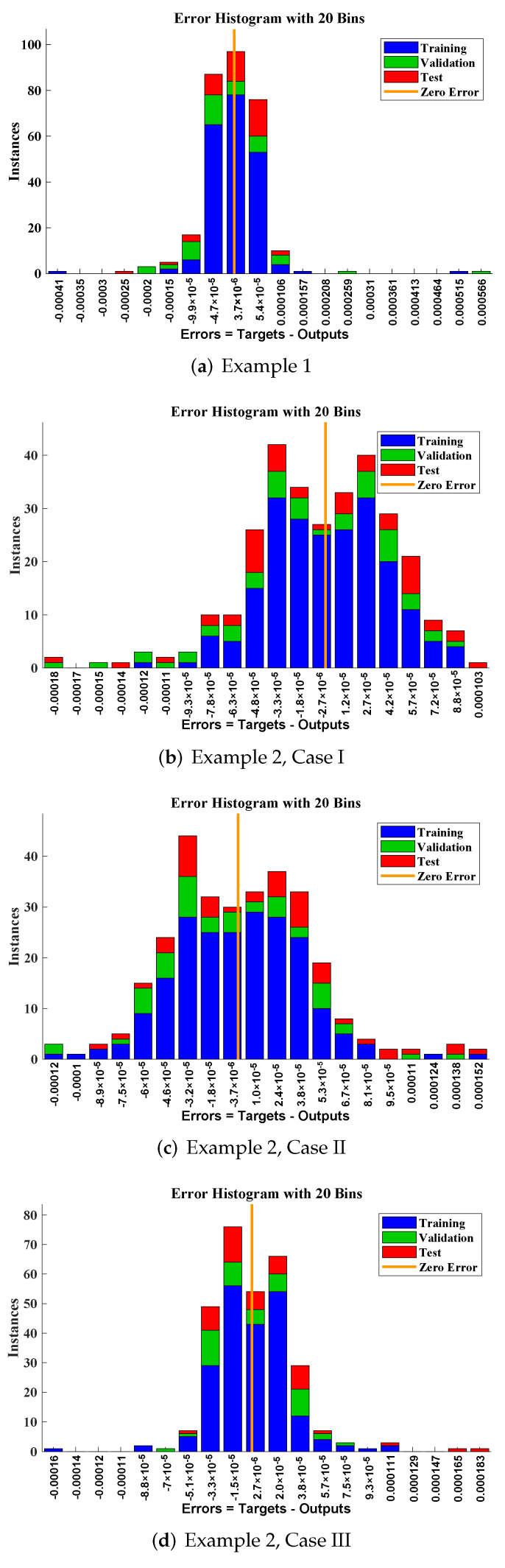

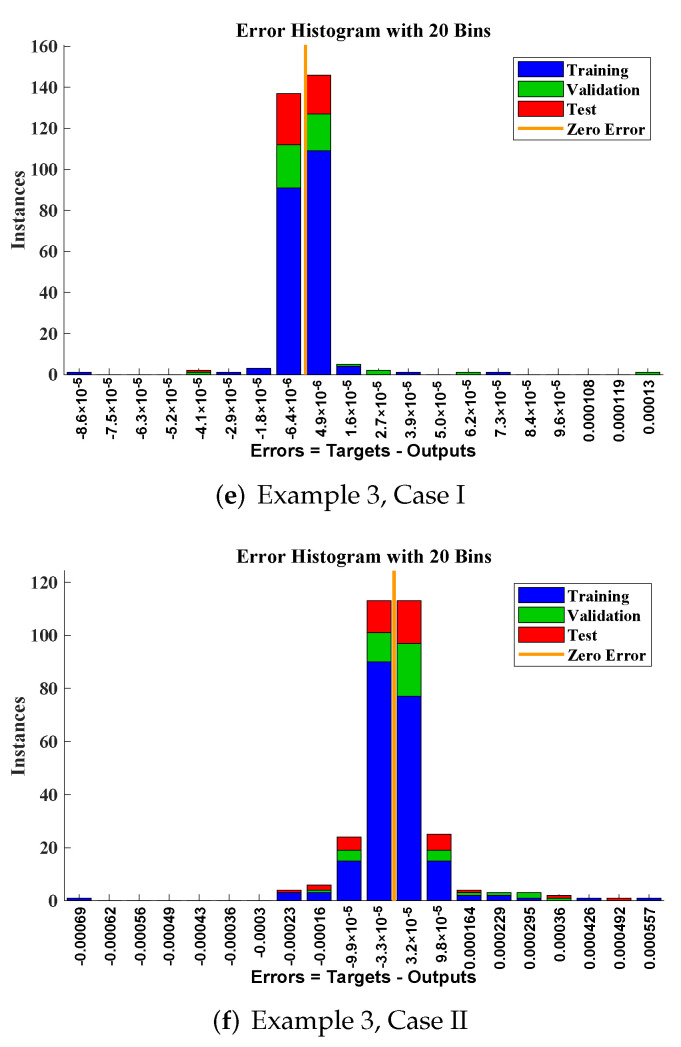
Error histograms, representing absolute errors between the reference solution and the approximated solution for different examples of the rolling motion of ships in random beam seas.

The design scheme is exploited to determine the fitting of approximate solutions, with the reference date set generated from the numerical solver using the RK-4 method. Curve fitting of the obtained solutions by LM-NNs for each example, with reference data of 301 points from 0 to 30, is shown in Figure 10. The performance of the objective function in terms of MSE to obtain best fitting is depicted in Figure 11. The best validated performance of MSE for different examples are $1.6631\times 10^{-8}$, $4.4759\times 10^{-9}$, $2.9493\times 10^{-9}$, $1.0449\times 10^{-9}$, $5.7951\times 10^{-10}$, and $1.1029\times 10^{-8}$, respectively. Figure 12 represents the absolute errors between the reference solution and the approximated solution by the LM-NN algorithm. It can be seen that the AE for different examples lies at around $10^{-4}$ to $10^{-6}$, $10^{-5}$ to $10^{-6}$, $10^{-4}$ to $10^{-6}$, $10^{-5}$ to $10^{-6}$, $10^{-5}$ to $10^{-6}$, and $10^{-4}$ to $10^{-5}$, respectively. These plots show the convergence, precision, and accuracy of LM-NNs for obtaining solutions to the mathematical model for the cubic damping of the rolling motion of ships.

**Figure 13:**
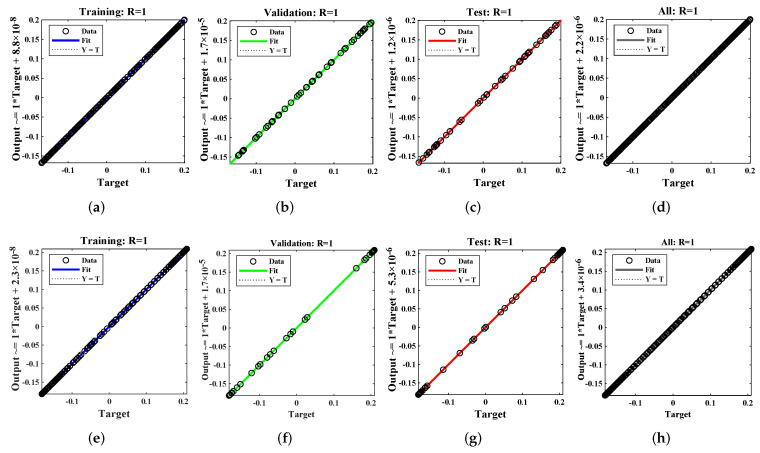

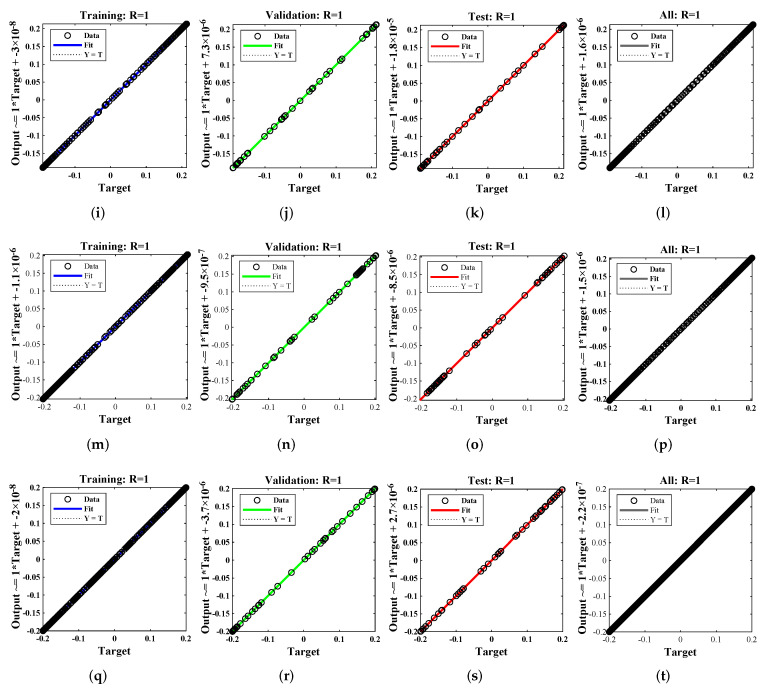
Regression analysis of Examples 1 and 2.

**Figure 14:**
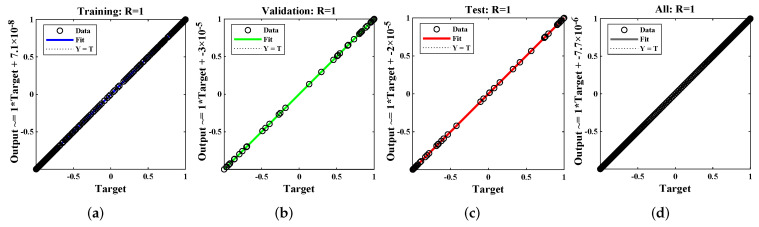
Regression analysis of Example 3.

**Figure 15:**
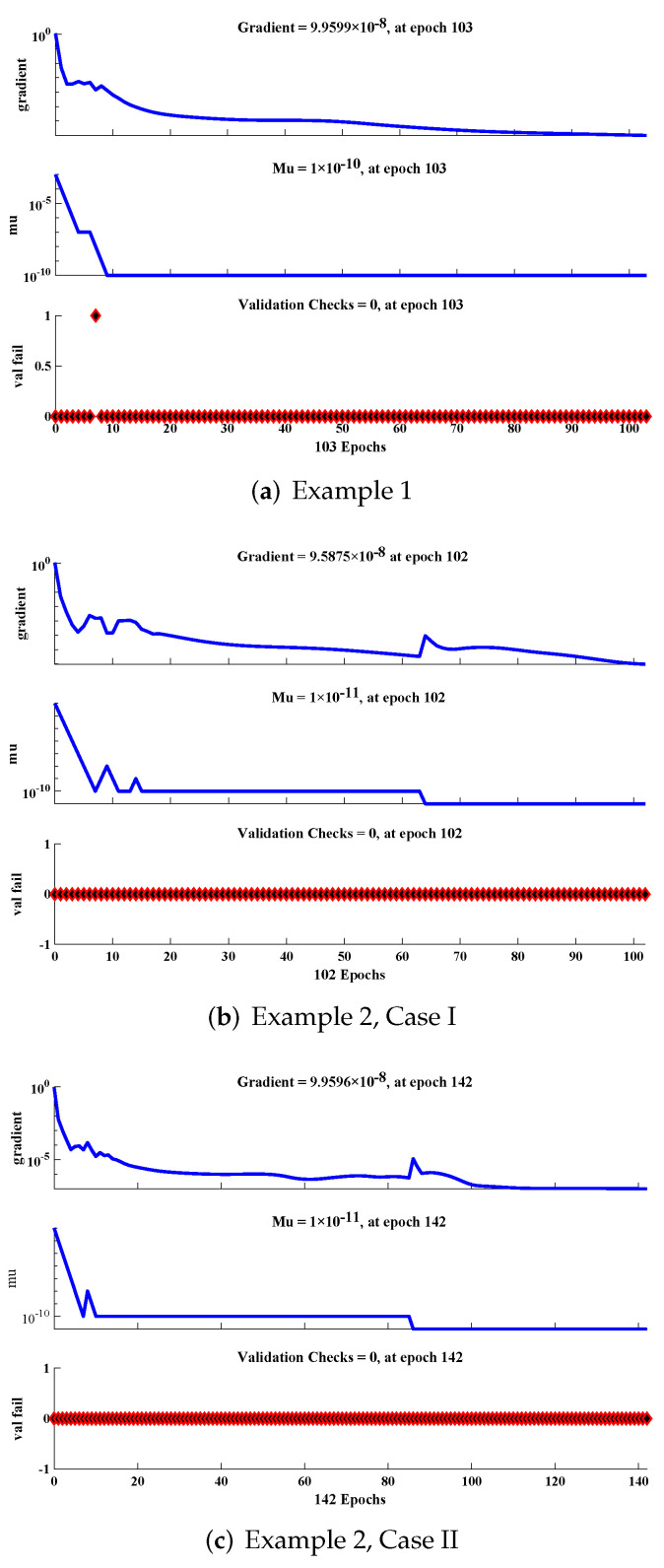

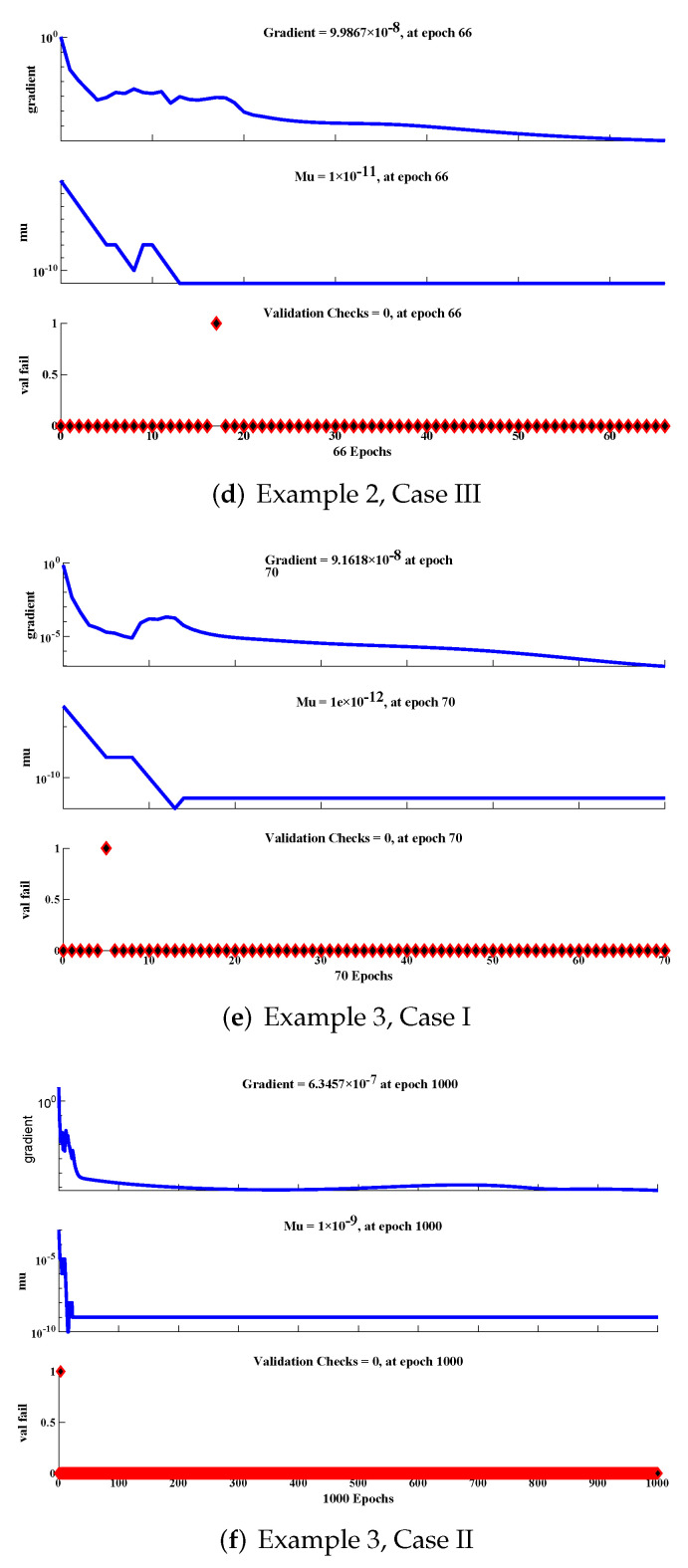
Training state of the design scheme for all examples of the rolling motion.

Regression values for each example are illustrated in Figure 13 and Figure 14. The correlation investigation was applied to study the regression analysis. The figures and Table 4 demonstrate that the value of “R” lies close to one, which reflects the perfect modeling of the solutions by the LM-NN algorithm. Figure 15 shows that values of the gradient and step size of mu for each example lies at around 9.9599 $\times 10^{-8}$, 9.5875 $\times 10^{-8}$, $9.9596\times 10^{-8}$, $9.9867\times 10^{-8}$, $9.1618\times 10^{-8}$, and $6.3457\times 10^{-7}$, with $10^{-10}$, $10^{-11}$, $10^{-11}$, $10^{-11}$, $10^{-12}$, and $10^{-9}$, respectively. Finally, all calculations and evaluations for this research were performed on an HP laptop EliteBook 840 G2 with intel(R) Core (TM) i5-5300 CPU @ 2.30 GHz, 8.00 GB RAM, 64 bit operating in Microsoft Windows 10 Education edition, running an R2018a version of MATLAB. The time taken by the CPU to solve such complex problems by the LM-NN algorithm are shown in Table 4.

## 6. Conclusions

This paper analyzes a mathematical model of the rolling motion of ships with nonlinear damping in random beam seas. To study the roll motion of ships under the effect of various forces and parameters, including angular frequency, strength of the nonlinearity coefficient, damping coefficient, viscous damping coefficients, and amplitude, we developed an intelligent soft computing technique based on artificial neural networks. A novel computing paradigm with a two-layer structure, the Levenberg-Marquardt (LM) algorithm and neural networks were utilized to calculate the approximate solution to the mathematical model by using a data set generated by numerical solvers such as the Runge–Kutta method or Adam’s method. Reference solutions of 70%, 15%, and 15% were utilized by the LM-NNs for the training, validation, and testing of the numerical examples. The results obtained by the proposed LM-NN algorithm were compared with those of the homotopy perturbation method (HPM), the Runge–Kutta method (RK-4), and the optimal homotopy analysis method (OHAM). Furthermore, to study the chaotic behavior of the rolling motion of ships, the mathematical model was solved by the LM-NNs to study the influence of variation on maximum slope $\alpha_{m}$. The results demonstrate that increasing the value of $\alpha_{m}$ causes an increase in the rolling angle of the ship. The system’s dispersion phenomena become apparent, and the rolling angle of the ship’s motion increases correspondingly. The technique’s performance is disclosed in terms of mean square error (MSE), regression $R^{2}$, error histograms, and performance evaluation. Extensive graphical and statistical results show the technique’s accuracy, precision, and robustness.

In the future, the designed scheme can be used to implement the solving of partial and fractional differential equations representing real-world problems.

## Figures and Tables

**Table 1:** Values of parameters involved in the mathematical model for the rolling of ships given in Equation (25).

Parameters	$n_{1}$	$n_{2}$	$n_{3}$	$m_{1}$	$m_{2}$	$A_{1}$	$A_{2}$
Values	3.24	−4.525	0.878	0.35	0.0222	0.504	−4.6656

**Table 2:** Approximate solutions obtained by the LM-NN algorithm for different examples of the rolling motion of ships in random beam seas.

	Example 1			Example 2			Example 3		
				**Case I**	**Case II**	**Case III**	**Case I**	**Case II**	
t	MHPM	Anaytical	LM-NN’s					OHAM	LM-NN’s
0	0.2	0.2	0.2	0.2	0.2	0.2	0.2	1	1
3	−0.166120	−0.191740	−0.191730	0.176480	0.176568	0.109021	0.118817	−0.338480	−0.334040
6	0.184868	0.174917	0.174914	−0.142790	−0.136060	−0.075050	−0.061160	−0.941280	−0.945690
9	−0.144550	−0.150800	−0.150820	−0.205370	−0.209320	−0.196250	−0.189810	0.775350	0.737937
12	0.157559	0.120891	0.120810	−0.191980	−0.190840	−0.137690	−0.163790	0.798647	0.767593
15	−0.112290	−0.086840	−0.086850	0.097787	0.091824	0.050338	−0.003150	−0.931210	−0.932550
18	0.121196	0.050406	0.050406	0.210881	0.210487	0.188166	0.160140	−0.398350	−0.395290
21	−0.072730	−0.013330	−0.013320	0.202523	0.207454	0.160958	0.191669	0.999319	0.999335
24	0.079358	−0.022720	−0.022720	−0.050090	−0.050840	−0.027350	0.067115	−0.27570	−0.267520
27	−0.029550	0.056298	0.056296	−0.210560	−0.203030	−0.179030	−0.113730	−0.950850	−0.957320
30	0.035768	−0.086160	−0.086160	−0.211550	−0.221450	−0.175970	−0.199900	0.749124	0.705858

**Table 3:** Results of velocity $\dot{x}\left(t\right)$ and acceleration $\ddot{x}\left(t\right)$ for different examples of the rolling motion of ships.

	Example 1				Example 2					Example 3		
			**Case I**		**Case II**		**Case III**		**Case I**		**Case II**	
*t*	$\dot{x}\left(t\right)$	$\ddot{x}\left(t\right)$	$\dot{x}\left(t\right)$	$\ddot{x}\left(t\right)$	$\dot{x}\left(t\right)$	$\ddot{x}\left(t\right)$	$\dot{x}\left(t\right)$	$\ddot{x}\left(t\right)$	$\dot{x}\left(t\right)$	$\ddot{x}\left(t\right)$	$\dot{x}\left(t\right)$	$\ddot{x}\left(t\right)$
3	−0.04120	0.17996	−0.05802	−0.05804	−0.05705	−0.05641	−0.04287	−0.00438	−0.05052	−0.01202	−0.78374	0.79843
9	−0.11206	0.14617	0.00643	−0.00788	0.00253	−0.00484	0.02417	0.02742	−0.01958	0.01839	0.37855	−0.36528
18	0.17082	−0.05193	−0.00528	−0.00236	0.00324	−0.00228	−0.03384	−0.02131	0.03741	−0.01583	0.72057	0.74845
27	−0.16067	−0.05433	−0.00085	0.01201	−0.00809	0.00652	0.02764	0.01857	−0.05169	0.01152	0.13380	0.80846

**Table 4:** Statistical analysis of the performance measures, including MSE, Gradient, mu, number of iterations, and time taken by the system to calculate the results.

			Mean Square Error							
Example	Case	Hidden Neurons	Training	Testing	Validation	Gradient	Mu	Epochs	Regression	Time (s)
1		30	$3.98\times 10^{-9}$	$1.66\times 10^{-8}$	$4.22\times 10^{-9}$	$9.96\times 10^{-8}$	$1.00\times 10^{-10}$	103	1	<1 s
2	I	30	$1.49\times 10^{-9}$	$4.48\times 10^{-9}$	$4.07\times 10^{-9}$	$9.59\times 10^{-8}$	$1.00\times 10^{-11}$	102	1	<1 s
2	II	30	$1.56\times 10^{-9}$	$2.95\times 10^{-9}$	$3.54\times 10^{-9}$	$9.69\times 10^{-8}$	$1.00\times 10^{-11}$	142	1	<1 s
2	III	30	$9.15\times 10^{-10}$	$1.04\times 10^{-9}$	$2.42\times 10^{-9}$	$9.99\times 10^{-8}$	$1.00\times 10^{-11}$	66	1	<1 s
3	I	30	$1.02\times 10^{-10}$	$5.80\times 10^{-10}$	$6.57\times 10^{-11}$	$9.16\times 10^{-8}$	$1.00\times 10^{-12}$	70	1	<1 s
3	II	30	$9.08\times 10^{-9}$	$1.10\times 10^{-8}$	$1.42\times 10^{-8}$	$6.35\times 10^{-7}$	$1.00\times 10^{-9}$	1000	1	1 s

## Data Availability

The data that support the findings of this study are available from the corresponding author upon reasonable request.
